# Large-scale wet-spinning of highly electroconductive MXene fibers

**DOI:** 10.1038/s41467-020-16671-1

**Published:** 2020-06-04

**Authors:** Wonsik Eom, Hwansoo Shin, Rohan B. Ambade, Sang Hoon Lee, Ki Hyun Lee, Dong Jun Kang, Tae Hee Han

**Affiliations:** 1 Department of Organic and Nano Engineering, Hanyang University, Seoul, 04763 Republic of Korea; 2 Research Institute of Industrial Science, Hanyang University, Seoul, 04763 Republic of Korea

**Keywords:** Materials science, Two-dimensional materials

## Abstract

Ti_3_C_2_T_*x*_ MXene is an emerging class of two-dimensional nanomaterials with exceptional electroconductivity and electrochemical properties, and is promising in the manufacturing of multifunctional macroscopic materials and nanomaterials. Herein, we develop a straightforward, continuously controlled, additive/binder-free method to fabricate pure MXene fibers via a large-scale wet-spinning assembly. Our MXene sheets (with an average lateral size of 5.11 μm^2^) are highly concentrated in water and do not form aggregates or undergo phase separation. Introducing ammonium ions during the coagulation process successfully assembles MXene sheets into flexible, meter-long fibers with very high electrical conductivity (7,713 S cm^−1^). The fabricated MXene fibers are comprehensively integrated by using them in electrical wires to switch on a light-emitting diode light and transmit electrical signals to earphones to demonstrate their application in electrical devices. Our wet-spinning strategy provides an approach for continuous mass production of MXene fibers for high-performance, next-generation, and wearable electronic devices.

## Abstract (web summary)

Large-scale production of fibers from two dimensional materials opens a pathway to promising applications. Here the authors report meter-long MXene fibers with high electrical conductivity that are fabricated via continuous wet spinning and demonstrated in electrical wires.

## Introduction

Two-dimensional (2D) nanosheets with fascinating properties are building blocks for potential applications^1,2^. Compared with their bulk counterparts, 2D nanomaterials are easy-to-assemble structures for nanoscale architectures that have appealing electronic, chemical, physical, and mechanical properties; a high specific surface area; and versatile surface chemistries^3–5^. To date, various 2D materials, such as graphene, hexagonal boron nitride (h-BN), graphitic carbon nitride (g-C_3_N_4_), transition metal dichalcogenides (TMDs), black phosphorus (BP), and transition metal oxides (TMOs), have garnered considerable attention, and many strategies have been proposed to develop them into macroscopic structures^6–10^. For example, significant advancements have been made regarding the development of macroscopic one-dimensional (1D) carbon-based fibers prepared from graphene oxides (GO)^11^. Graphene-related fibers have gained considerable interest because of their versatile functionalities, such as lightweight, mechanical flexibility, bendability, stretchability, and the ability to be woven into textiles for the next generation of smart electronic gadgets^12,13^. In particular, to realize the macroscopic assembly of 2D nanosheets into fiber structures, the wet-spinning process, which utilizes the phase change ability of highly concentrated colloidal dispersions (i.e., \ in the liquid state) to transform into gel-fiber assemblies and solid fibers in a coagulation bath, has been demonstrated as a versatile pathway for the long and continuous mass production of fibers^11,14^. Notably, understanding the molecular interaction between sheets and systematic studies on the parameters of the coagulation process are crucial to achieving fiber formation from individual colloidal particles.

Ti_3_C_2_T_*x*_ MXene is composed of transition metal nitrides and carbides (MXenes) and has been extensively explored as an emerging family of 2D materials because of its excellent electrical–thermal conductivity, mechanical, and chemical properties, and wide range of potential applications^15–19^. MXenes have the general configuration of *M*_*n*+1_*X*_*n*_*T*_*x*_, in which *M*, *X*, and *T* represent transition metals, carbon/nitrogen, and surface terminal functionalities, such as O, F, and OH, respectively. MXenes are typically obtained as sheet materials with a nanoscale thickness via the delamination of the *MAX* (*M*_*n*+1_*AX*_*n*_) phase^16^. Very recently, several researchers have attempted to fabricate MXene-based fibers using wet-spinning and electrospinning with MXene/polymer blend dope solutions and MXene/rGO for coassembly^20,21^. However, the intrinsically high electrical conductivity of pure Ti_3_C_2_T_*x*_ MXene (up to 9880 S cm^−1^ for spray-cast films) is lower than that of MXene composites with reduced GO (rGO) (72–290 S cm^−1^)^21,22^, CNT fiber (26 S cm^−1^)^23^, and PEDOT:PSS (1489 S cm^−1^)^24^, showing that the conductivity of MXenes is not fully utilized in the fiber form. The crucial challenge regarding wet-spinning for pure MXene fibers is the weak self-supporting organization because of poor interlayer interaction between the relatively small MXene sheets. In addition, a low concentration of dispersion has made it challenging to process MXene directly into a 1D fiber form.

**Fig. 1:**
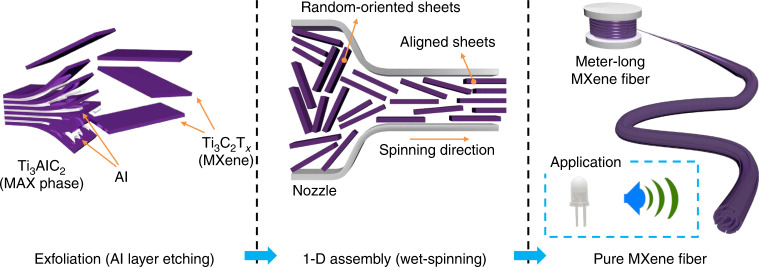
Schematic illustration of the reconstruction of MXene single layers into MXene fibers. Ti_3_AlC_2_ was exfoliated to Ti_3_C_2_T_*x*_ by etching the Al layer. The MXene was highly concentrated in the aqueous dispersion and assembled into a fiber, which was aligned in the axial direction by the wet-spinning process. The obtained pure MXene fiber was used for electric energy and signal transfer applications.

Herein, we report a straightforward and reliable synthetic route for continuously controlled fabrication of additive/binder-free, composite-free, entirely pure 1D MXene fibers with high electrical conductivity by a wet-spinning assembly (Fig. 1). The dispersion with a relatively large Ti_3_C_2_T_*x*_ MXene sheet (the average size and aspect ratio were ~5.11 μm^2^ and 1600, respectively) at a high concentration (25 mg mL^−1^) demonstrated highly stable colloidal properties in a lyotropic liquid-crystalline phase. The wet-spinning of the 2D MXene spinning dope successfully produced flexible meter-long continuous MXene fibers with an ultrahigh electrical conductivity of 7713 S cm^−1^. The MXene fibers that exhibited excellent performance were used for the electrical wires to switch on an LED light and wires to transmit electrical signals to earphones. Furthermore, the MXene fibers showed high flexibility and excellent mechanical properties. The wet-spinning strategy reported in this work suggests a method for the continuous mass production of MXene fibers, which indicates that they are promising candidates for high-performance, flexible, portable, and wearable electronics. The development of nanoscale properties on the macroscopic level using a scalable assembly represents progress toward the practical application of these extraordinary 2D materials.

## Results

### Synthesis and characterization of Ti_3_C_2_T_*x*_ MXene sheets

**Fig. 2:**
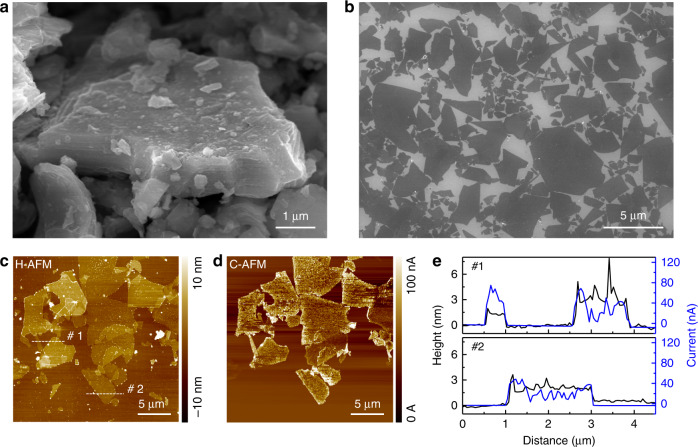
Synthesis and identification of Ti_3_C_2_T_*x*_ (MXene). Morphology of **a** MAX-phase particle and **b** MXene single layer coated on a SiO_2_ wafer. **c** AFM and **d** C-AFM images of MXene single sheets in the same area. **e** Height and current-line profile of lines #1 and #2 in **c**.

A MAX-phase (Ti_3_AlC_2_) powder with graphite-like stacked-layer structures was observed in images obtained via scanning electron microscopy (SEM) (Fig. 2a and Supplementary Fig. 1a). MXene (Ti_3_C_2_T_*x*_) sheets were obtained by selectively etching the Al from a Ti_3_AlC_2_ powder using LiF and HCl, as reported previously^25^. SEM images of fully exfoliated MXene monolayers showed an average lateral size of 2.26 ± 0.95 μm (Fig. 2b and Supplementary Fig. 1b, c). The height profile obtained via atomic force microscopy (AFM) mapping revealed that the MXene sheets had a height of 1.35–1.81 nm, which corresponded to a single layer of the MXene, implying the successful exfoliation of the sheets (Supplementary Fig. 1d and e)^26^. The folded MXene was identified as a double layer according to the AFM height profile, which showed a height of 3.31–3.72 nm; this agrees with the results of previous reports^26^. Conductive atomic force microscopy (C-AFM) clearly showed that the MXene sheets were very electrically conductive (Fig. 2d, e). The prepared MXene monolayer was also observed using transmission electron microscopy (TEM), and highly crystalline lattice fringes with a lattice spacing of 0.26 nm corresponding to the Ti_3_C_2_T_*x*_ (100) plane were clearly observed in the HR-TEM images. The selected area electron diffraction (SAED) pattern confirmed that the MXene sheets had a typical hexagonal symmetry (Supplementary Fig. 2a and b). The XRD pattern and the atomic percent of MXene confirmed the complete etching of the Al layer (Supplementary Fig. 2c and Supplementary Table 1)^27^. The corresponding elemental maps confirmed the uniform distribution of all elements, suggesting that the surface of the MXene sheet contained oxygen and fluorine as termination groups (Supplementary Fig. 2d).

**Fig. 3:**
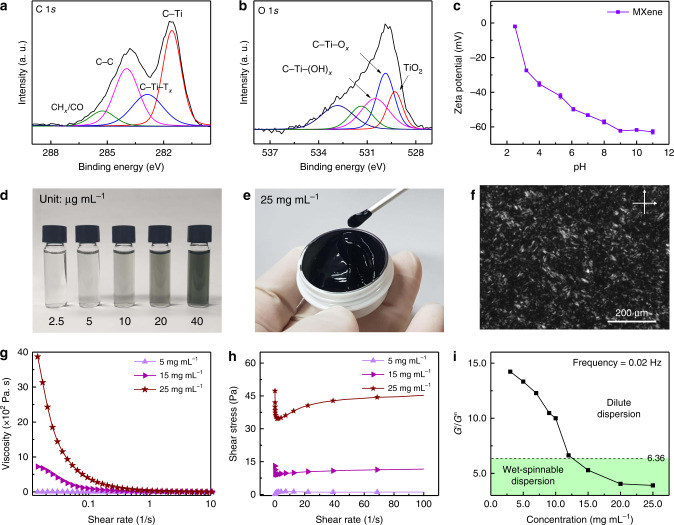
Dispersibility and spinnability of MXene nanosheets in liquid-crystalline dispersion. **a** C 1*s* and **b** O 1*s* XPS spectra of MXene obtained from the dispersion. **c** Zeta potential of the MXene as a function of pH in the aqueous dispersions at a concentration of ~0.05 mg mL^−1^. **d** Optical image of diluted MXene dispersions with concentrations of 0.0025, 0.005, 0.01, 0.02, and 0.04 mg mL^−1^ at 755 nm. **e** Optical image of the concentrated MXene LC dispersion (25 mg mL^−1^). **f** POM image of the MXene dispersion (20 mg mL^−1^) that exhibited optical birefringence. **g** Steady shear rheological properties of the MXene LC dispersions with various concentrations (1–25 mg mL^−1^). **h** Shear stress of the MXene dispersion as a function of shear rate. **i** *G*′/*G*″ ratio of the MXene dispersion as a function of concentration. The green region under *G*′/*G*″ = 6.36 indicates the region for the wet-spinning of the MXene dispersion at a specific shear rate (0.02 Hz).

The chemical functionalities of the exfoliated MXene sheets were further examined via X-ray photoelectron spectroscopy (XPS). The deconvoluted C1*s*, O1*s*, and Ti2*p* XPS peaks demonstrated that inherent termination groups, such as C–Ti–T_*x*_, C–Ti–(OH)_*x*_, and C–Ti–O_*x*_, existed on the surface of the MXene and were likely introduced during the Al etching of the MAX crystals (Fig. 3a, b, Supplementary Fig. 2a–c). Notably, these surface functionalities are important for the formation of a stable dispersion in an aqueous medium^28^. The negative surface charge values increased with the pH of MXene, owing to the ionizable surface termination groups, suggesting strong electrostatic repulsion between the adjacent sheets (Fig. 3c)^29,30^. The apparent dispersibility of the MXene was observed at various concentration ranges (Fig. 3d), and no sediment was formed on the bottom of the vials at different MXene concentrations. The absorbance of the MXene dispersion was examined, as shown in Supplementary Fig. 3d. In the inset of Supplementary Fig. 3d, a linear relationship between the UV absorbance and concentration of MXene sheets was observed, confirming the stability of the dispersed state^31^.

### Rheological properties of MXene ink

At a high concentration (25 mg mL^−1^), the MXene dispersion formed a viscous ink with a viscosity of 3.87 × 10^3^ Pa s and without aggregates and phase separation of solid particles and dispersing media (Fig. 3e)^28,32^. Based on Onsager’s theoretical prediction model, MXene sheets can exhibit lyotropic liquid-crystalline properties from ~16 mg mL^−1^ ^33^. As shown in Fig. 3f, the MXene dispersion (25 mg mL^−1^) also exhibited birefringence between two crossed polarizers, indicating the formation of a liquid-crystalline phase as a result of local orientation that did not aggregate. As is often observed in complex fluid systems containing rigid polymer chains, the viscosity of the MXene increased with the concentration and decreased with an increase in the shear rate (Fig. 3g)^34–36^. Furthermore, the shear stress of the MXene dispersion significantly decreased at the initial stage and then gradually increased with the shear rate (Fig. 3h), suggesting that the randomly oriented MXene sheets became arranged as a result of the shear-induced deformation^10^. A decrease in the shear stress was clear in concentrated dispersions (higher than 15 mg mL^−1^). Notably, the ratio of the storage modulus to the loss modulus (*G*′/*G*″) of a dispersion can be used as an indicator for the spinnability of liquid-crystalline 2D colloidal dispersions^37^. Wallace et al. reported that the wet-spinning of GO dispersions is achievable when the value of *G*′/*G*″ is between 1.80 and 6.36 at an angular rate of 0.02 Hz. Gogotsi et al. also expected that highly concentrated MXene dispersions can be wet-spun into fibers by following this relation^35^. Similarly, we found that the spinnability of an MXene dispersion could be predicted using the *G*′/*G*″ value of the MXene. Experimentally, at 5 mg mL^−1^, the MXene dispersion herein was not capable of forming fibers because of the weak gel strength, and the value of *G*′/*G*″ was 13.33 (Supplementary Fig. 4). When the *G*′/*G*″ value of the MXene was 6.64 at 12 mg mL^−1^, the fiber was not stably formed herein, but MXene dispersions with over 15 mg mL^−1^ (the *G*′/*G*″ value was 5.29 at 15 mg mL^−1^) were successfully fabricated into MXene fibers (Fig. 3i).

### Wet-spinning of pure MXene fibers

**Fig. 4:**
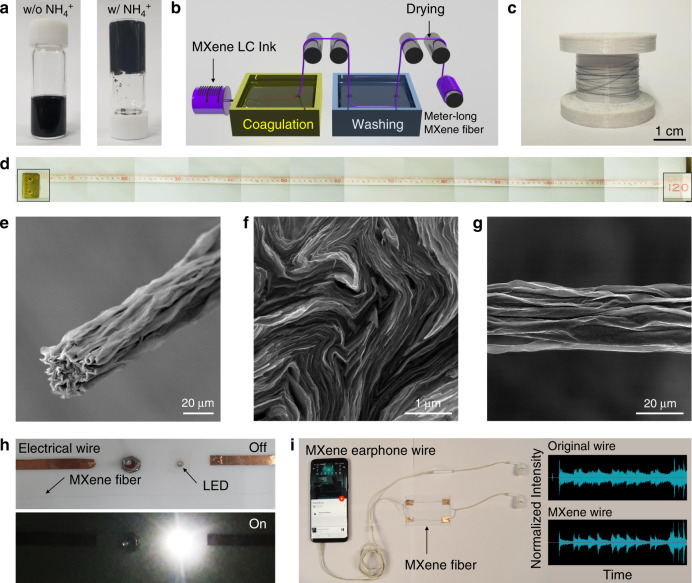
Wet-spinning of the pure Ti_3_C_2_T_*x*_ MXene fiber. **a** Gelation of the Ti_3_C_2_T_*x*_ MXene dispersion by NH_4_^+^ ions. The sol–gel transition was identified via the vial-inversion method. **b** Schematic illustration of the wet-spinning process of the Ti_3_C_2_T_*x*_ MXene fiber. **c** Meter-long Ti_3_C_2_T_*x*_ MXene fiber wound on the bobbin. **d** Continuous wet-spinning allowed fabrication of over 1 m (1.2 m) of the Ti_3_C_2_T_*x*_ MXene fiber. SEM images of Ti_3_C_2_T_*x*_ MXene fiber: **e** overall, **f** in cross-section, and **g** side-section views. Ti_3_C_2_T_*x*_ MXene fiber applied as **h** an electrical wire and **i** an earphone wire.

Note that the colloidal stability of MXene sheets can be considerably affected by salts. The role of NH_4_ ions in the gelation of MXene dispersions was confirmed by the vial inversion method (Fig. 4a)^14^. Indeed, similar to the behavior of graphene and other 2D materials, the high degree of exfoliation/delamination and gelation of MXenes is essential for continuous fiber fabrication. The prepared MXene liquid crystal dispersion was extruded into a coagulation solution with NH_4_ ions and then washed in a water bath through a reel to produce continuous fibers using a simple wet-spinning method (Fig. 4b and Supplementary video). The extruded MXene did not form gel fibers without NH_4_ ions (Supplementary Fig. 5). Finally, the fibers were dried in air for 24 h and formed uniform, long, continuous MXene fibers oriented in the axial direction. The meter-long MXene fibers produced on a large scale by continuous spinning were wound onto a bobbin (Fig. 4c). The extruded 100% pure MXene fibers that were longer than 1 m were stable with continuous spinning (Fig. 4d). The cross-section of the MXene fibers showed a lamellar structure with highly compact nanosheets (Fig. 4e–g). The rugged morphology on the side of the fibers indicated that drying and shrinking occurred (Fig. 4g). Highly conducting MXene fibers were used for an electrical application that involved successfully switching on a white light-emitting diode (LED) light (Fig. 4h). Furthermore, the MXene fibers replaced commercially used wires and were integrated into earphone wires to transmit electrical signals (Fig. 4i, Supplementary audio).

### Performances of MXene fiber

**Fig. 5:**
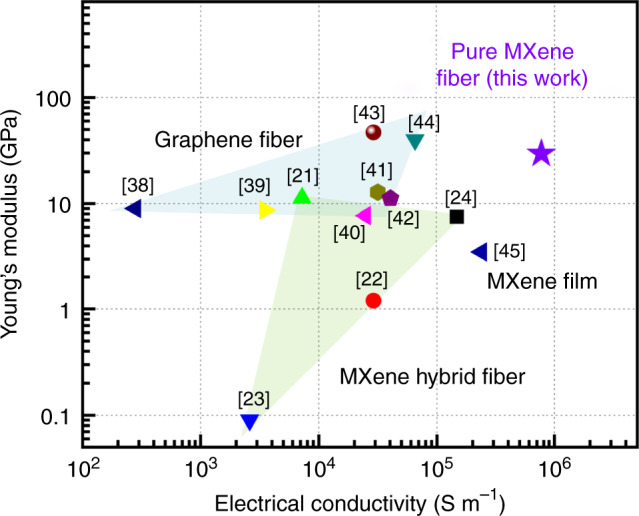
Comparison of electrical conductivity and Young’s modulus. The MXene fiber is compared with previous graphene fibers and MXene hybrid fibers.

Figure 5 compares the electrical conductivity and Young’s modulus of our fabricated MXene fibers with those of MXene hybrid fibers and graphene fibers fabricated in previous studies (Supplementary Fig. 6 and Supplementary Table 2)^21–24,38–45^. From the Ashby plot, it is clear that our wet-spun pure MXene fibers are superior to the other considered fibers in terms of electrical conductivity and Young’s modulus. The electrical conductivity of the MXene fibers (7713 S cm^−1^) was almost 107 and 27 times higher than that of MXene/graphene hybrid fibers (72.3 and 290 S cm^−1^, respectively)^21,22^ and five times higher than that of MXene/PEDOT:PSS fibers (1490 S cm^−1^)^24^, which were reported previously. Furthermore, the conductivity of the MXene fibers in this work was ~12–220 times higher than that of the graphene fibers^38–44^. In addition, the pure MXene fibers were 3.2 times more conductive than reported MXene films at the macroscopic scale, implying that the MXene fibers had a well-constructed structure^45^.

## Discussion

We effectively developed pure Ti_3_C_2_T_*x*_ MXene fibers that were free of additives/binders or composites via a straightforward, continuous, large-scale, wet-spinning strategy. The large Ti_3_C_2_T_*x*_ MXene sheets had an excellent dispersion at a high concentration of 25 mg mL^−1^ and demonstrated liquid-crystalline patterns and rheological properties of lyotropic liquid crystals. The Ti_3_C_2_T_*x*_ MXene fibers fabricated by a wet-spinning method herein exhibited a very high electrical conductivity of 7713 S cm^−1^, and flexible, continuous, meter-long MXene fibers were successfully fabricated. Considering these outstanding properties, we comprehensively integrated the Ti_3_C_2_T_*x*_ MXene fibers in electrical wires for switching on an LED light and transmitting electrical signals to earphones to demonstrate the application of the fibers in miniaturized portable devices. Therefore, we believe that our wet-spinning strategy for continuous mass production of pure Ti_3_C_2_T_*x*_ MXene fibers offers a way to exploit the original nanoscale potential of MXenes at the macroscopic scale. In addition, the approach herein advances the use of a large family of MXenes in next-generation flexible, portable, and wearable miniaturized electronic devices.

## Methods

### Materials

The layered ternary carbide (Ti_3_AlC_2_) MAX-phase powders were purchased from Carbon-Ukraine Ltd. (particle size < 200 μm, Ukraine). The chemicals, including hydrochloric acid (HCl), lithium fluoride (LiF), ammonium chloride (NH_4_Cl), and ammonium hydroxide (NH_4_OH), were purchased from Sigma-Aldrich Co (St. Louis, MO, USA). Deionized water (DIW) was obtained using a water-purification system (Direct Q3) purchased from Millipore (Bedford, MA, USA).

### Synthesis of the Ti_3_C_2_T_*x*_ MXene

The Ti_3_C_2_T_*x*_ MXene was obtained from Ti_3_AlC_2_ precursors by modifying a previously reported method^25^. A quantity of 2 g of LiF was dissolved into 40 mL of a 9 M HCl solution in a reactor. The solution was stirred for 30 min at 35 °C. After slowly adding 2 g of the Ti_3_AlC_2_ (MAX phase) powder, the mixture was stirred in an argon atmosphere for 24 h. The Al layer in the MAX phase was etched to exfoliate the Ti_3_C_2_T_*x*_. Then, 40 mL of the obtained solution was divided into 20 mL conical tubes and diluted with water (20 mL). To separate the MXene dispersion from the acid, it was washed with water using a centrifuge until the pH reached 6. As the pH of the solution was almost neutral, the MXene in the dispersion did not sink well through centrifugation due to an increase in the negative zeta potential of their surface. The washed solution (pH 6) was centrifuged repeatedly to purify the MXene sheets and to concentrate the MXene dispersion. The obtained solution was sealed with parafilm and stored at ~5 °C.

### Wet-spinning of the pure Ti_3_C_2_T_*x*_ (All-MXene) fibers

The MXene dispersion was placed in a syringe and extruded through the nozzle (diameter = 210 μm) into the prepared coagulant. The coagulate solution was a mixture of NH_4_Cl (50 g), NH_4_OH solution (20 mL), and DIW (1000 mL). The MXene dispersions were extruded at a velocity of 7 mL h^−1^. The extruded MXene fibers in the coagulate solution were transferred to a washing bath by rollers. The washed fibers were dried in air and then stored in a dry chamber.

### Characterization

The concentration of the concentrated dispersion was defined by cross-validation with the Beer–Lambert Law using UV–vis spectroscopy (Lambda 650S, Perkin Elmer, USA) and directly measuring the mass of the powder per unit volume of dispersion using an ultra-micro balance (XPR2U, Mettler-Toledo GmbH, Greifensee, Switzerland). The dimensions of the MXene sheets and the morphologies of the MXene fibers were characterized using SEM (S4800, Hitachi, Japan) at 15 kV and 10 μA without Pt sputtering. The topography of the MXene sheets was observed via AFM (XE-70, Park Systems, Korea) in tapping mode. The measured data were processed using a data processing and analysis software (XEI, Park Systems). The topography and a current image of the MXene nanosheets on the Si wafers were obtained simultaneously during the C-AFM scan. In this study, we conducted current measurements on the in-plane surface, which was consistent with the direction of the carrier transport parameter measurements. A cantilever (CDT-CONTR, Park systems) was used, and the measurement was performed with a fixed bias of 10 V. The cantilever had a resonance frequency of ~20 kHz, a spring constant of 0.5 N m^−1^, and a tip radius between 100 and 200 nm. All measurements were performed at room temperature (~25 °C) and in ambient conditions. The synthesized MXene single layer was characterized using HR-TEM (JEM-2100F, JEOL, Japan). The terminal group and the chemical state of the MXene were analyzed via XPS (Theta probe, Thermo Scientific, UK) with monochromatic Al Kα radiation. The XPS spectra were analyzed using Xpspeak41 software. The rheological properties of the MXene dispersion were measured using a rheometer (MCR 501, Anton Paar, Austria) under both steady shear and dynamic oscillatory conditions. The viscoelastic properties of the Ti_3_C_2_T_*x*_ dispersion were investigated by measuring the storage and loss modulus as a function of frequency from 0.1 to 1000 rad/s. During the frequency sweep, the strain amplitude was maintained at 0.1% (a gap of 1 mm, 25 °C)^35^. An optical image of the meter-long MXene fibers with a ruler was obtained by joining several close-up images taken vertically from above the fibers because the fibers were too thin for an image to be obtained from far away. The tensile properties of the MXene fibers prepared from different concentrations of dispersion were investigated using a universal testing machine (5966, Instron, USA) equipped with a 10 N load cell that operated at a crosshead speed of 2.5 mm min^−1^ and had a gauge length of 25 mm; the tensile measurements of single ultrafine fibers reported in a previous study were used as a reference^14^. The fibers were loaded on a specific rectangular frame. The mechanical strength of the MXene fibers was calculated by dividing the force by the cross-sectional area. The electrical conductivity of the MXene fibers was measured using a multimeter (DMM 7510 1/2, Keithley Instruments, USA) via the four-point probe method. Four electrodes were separated by a distance of 0.4 mm. Since the conductivity of MXene fibers is highly affected by the atmospheric humidity, the measurement was made in a dry chamber. The conductivity (*ρ*) of a single fiber was calculated according to Eq. (1) as follows^46^:

1 $$\rho = \frac{{\pi d^2R}}{{4L}}$$

where *d* is the diameter, *R* is the electrical resistance, and *L* is the length of the fiber. The diameter of the fiber was calculated based on the cross-sectional area measured in the scanning electron microscope (Supplementary Fig. 7).

### Prediction of phase change using the Onsager model

To predict the critical value of the anisotropic phase, we measured the distribution of the MXene monolayer lateral sizes (Supplementary Fig. S1c) and found a reasonable agreement with the Onsager model for lyotropic liquid crystals in a disk formulation. The concentration (mass fraction) at the isotropic–nematic phase transition (*C*, the critical value) was calculated according to Eq. (2) as follows^33^:

2 $$\frac{d}{I} \approx 5\frac{{\rho _{\mathrm{{{MXene}}}}}}{{\rho _{\mathrm{{{suspension}}}}}}C^{ - 1}$$

where *d* and *l* are the lateral size and thickness of the MXene nanosheets, respectively; *ρ*_MXene_ is the true material density (5.2 g cm^3^)^47^; and *C* is the concentration (mass fraction) at the isotropic–nematic phase transition.

## Supplementary information

Supplementary Information

Peer Review File

Supplementary Audio

Supplementary Movie

## Data Availability

The datasets generated during and/or analyzed during the current study are available from the corresponding author on reasonable request. Source data are provided with this paper.

## Source data

Source Data
