# Supplementary material for: Large-scale wet-spinning of highly electroconductive MXene fibers
Source: Nat Commun. 2020 Jun 4;11:2825. doi: 10.1038/s41467-020-16671-1 (PMC7272396; doi:10.1038/s41467-020-16671-1)
Supplement: Supplementary file 1 — Supplementary Information [file 41467_2020_16671_MOESM1_ESM.pdf]

Supplementary Information

Large-scale Wet-spinning of Highly Electroconductive MXene Fibers

Eom et al.

Supplementary Table 1. Atomic percent of components in the MXene ($Ti_3C_2T_x$) and MAX phase (Ti_3AlC_2).

Sample	Ti 2p	Al 2p	C 1s	O 1s	F 1s
Ti_3AlC_2	8.48	11.24	36.41	43.75	0.12
Ti_3C_2	29.94	0.11	39.09	17.92	12.94

Supplementary Table 2. Comparison of the performance of different $Ti_3C_2T_x$ MXene fibers.

Sample	Electrical conductivity (S cm^{-1})	Tensile Strength (MPa)	Tensile Elongation (%)	Young's Modulus (GPa)	Toughness (MJ m^{-3})	Reference
Pure MXene Fiber	7713 ± 110	63.9 ± 13.1	0.22 ± 0.05	29.6 ± 5.1	0.091 ± 0.011	This study
MXene/rGO fiber	72.3	132.5	2.9	11.3		[1]
MXene/graphene fiber	290	12.9	3.4	1.2		[2]
MXene/CNT yarn	26	27	15.1	0.09		[3]
MXene/PEDOT:PSS fibers	1489.8	58.1	1.1	7.5		[4]

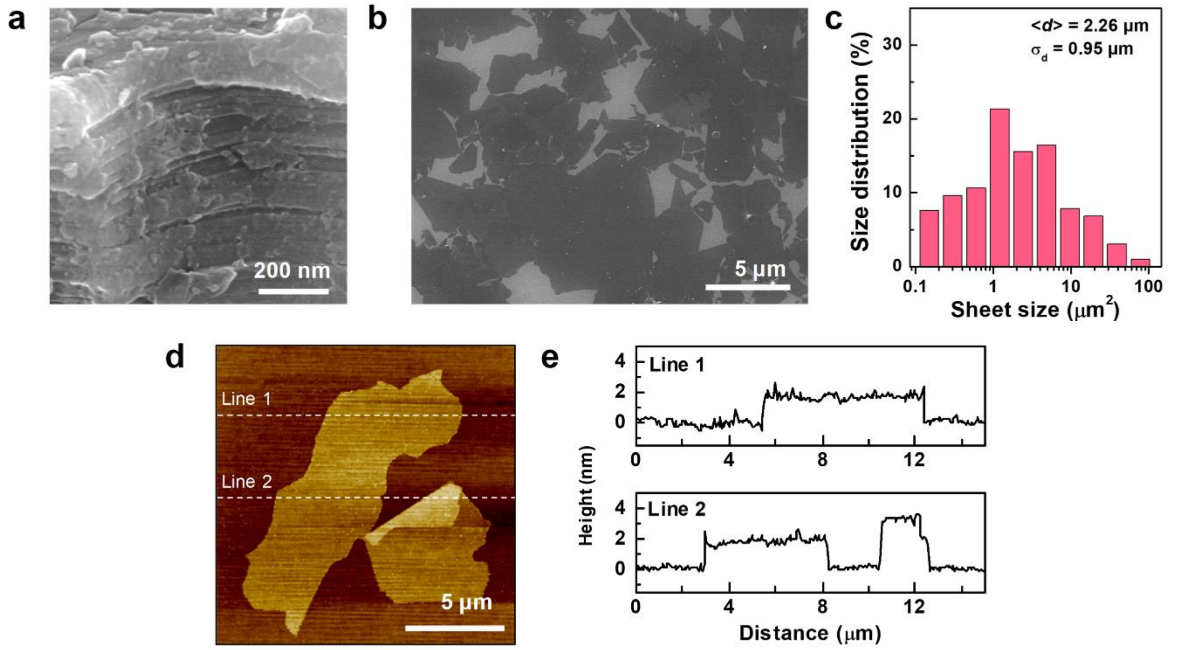

Supplementary Figure 1. SEM image of **a** layered MAX powder and **b** fully exfoliated MXene sheets. **c** Lateral size distribution of MXene sheets. The distribution of the sheet sizes was obtained by measuring 635 sheets in SEM images. The mean lateral size of the sheets was 2.26 ± 0.95 µm. **d** AFM image of MXene single-layer sheets coated on a SiO_2 substrate and **e** line profile of the MXene sheets.

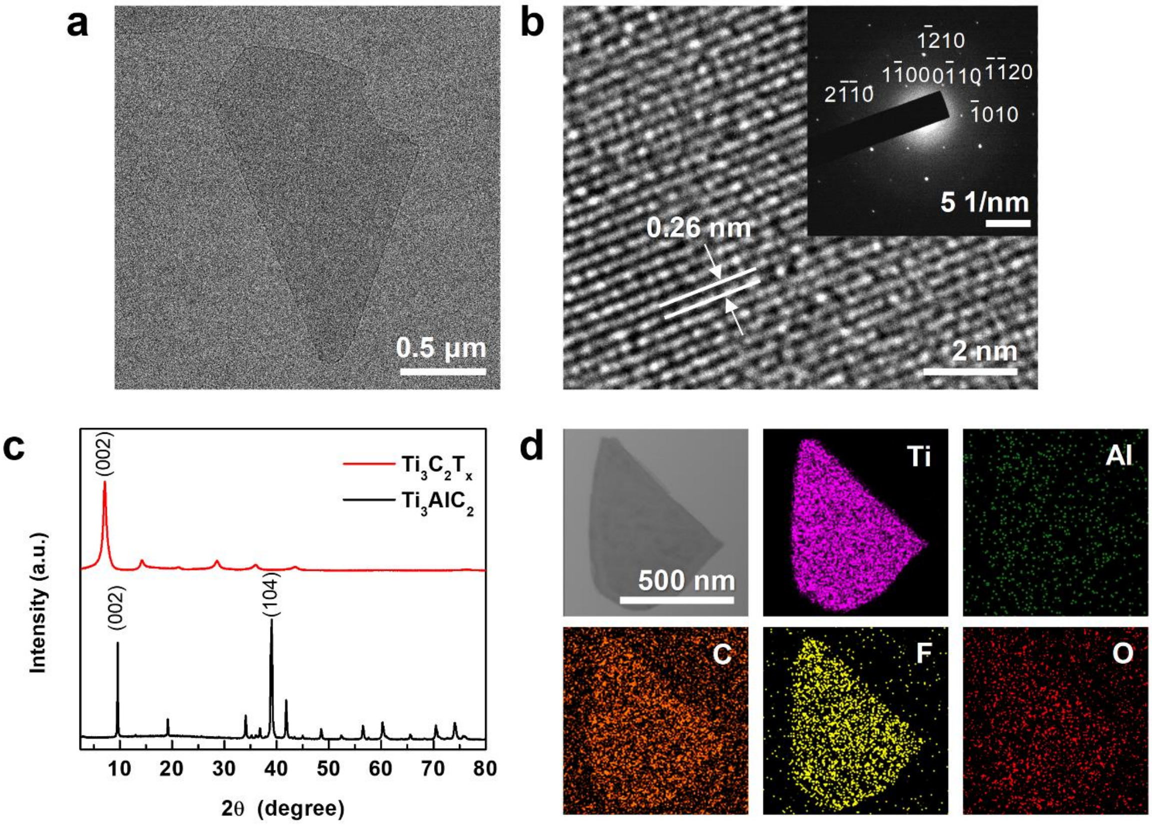

Supplementary Figure 2. **a** Low-resolution TEM image of isolated MXene single layer and **b** HR-TEM image showing lattice fringes of an MXene sheet (inset image shows the SAED pattern of the MXene sheet). **c** XRD patterns of MXene ($Ti_3C_2T_x$) and MAX phase (Ti_3AlC_2). **d** EDX elemental mapping of the MXene single layer.

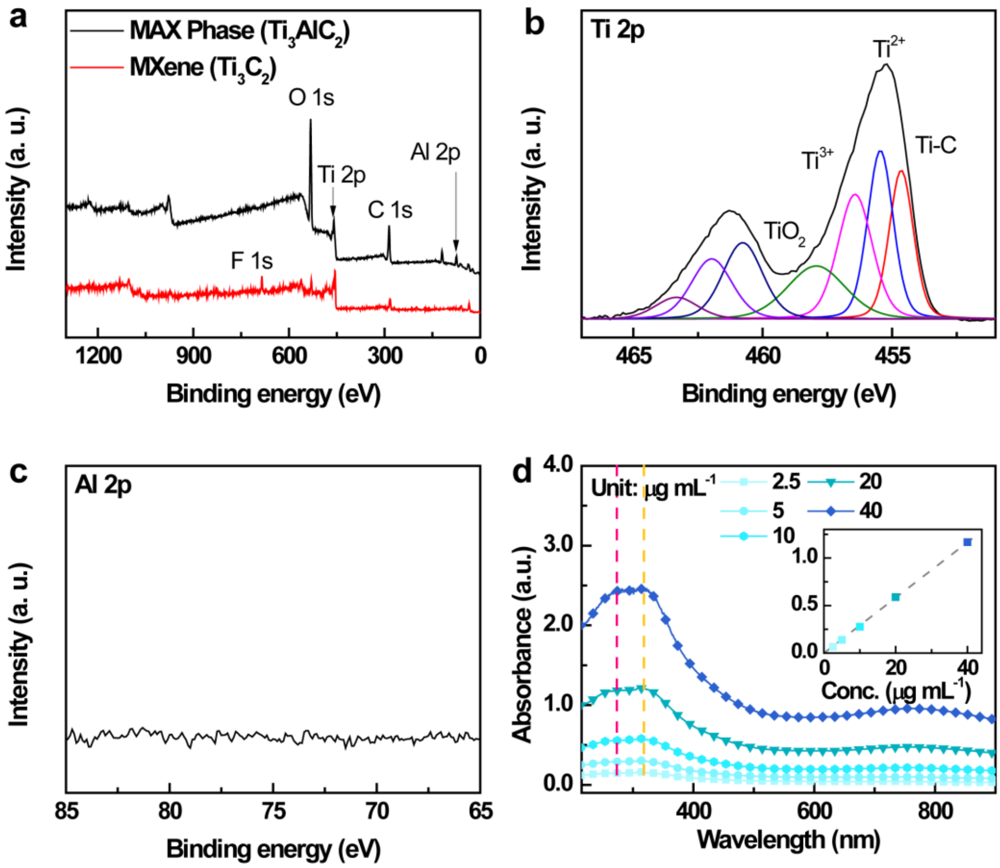

Supplementary Figure 3. **a** XPS survey and XPS high-resolution spectra of **b** Ti 2p and **c** Al 2p of the MXene. **d** UV-vis spectroscopy of the diluted $Ti_3C_2T_x$ MXene dispersion. (inset: absorbance of the diluted $Ti_3C_2T_x$ MXene dispersion with concentrations of 2.5, 5, 10, 20, and 40 μg mL^{-1} at 755 nm.)

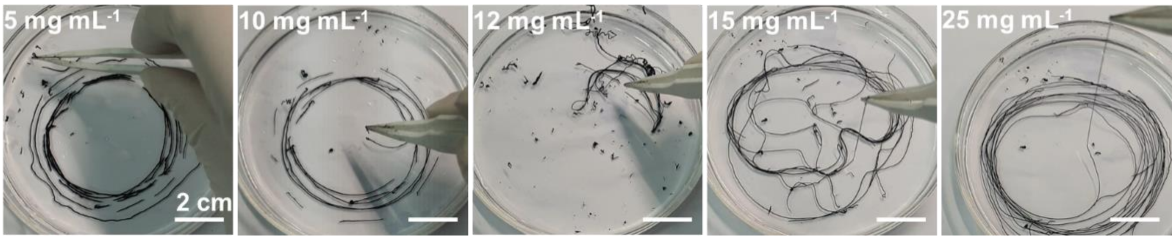

Supplementary Figure S4. Digital photos that demonstrate the spinnability of the MXene liquid-crystalline dispersion as a function of the dope concentration. When the elastic modulus (G′) dominated, the MXene dispersion was suitable for fiber fabrication and dispersion and could maintain its given shape. Above 15 mg mL^{-1}, the MXene gel fibers could be removed from the coagulation bath while retaining their shape.

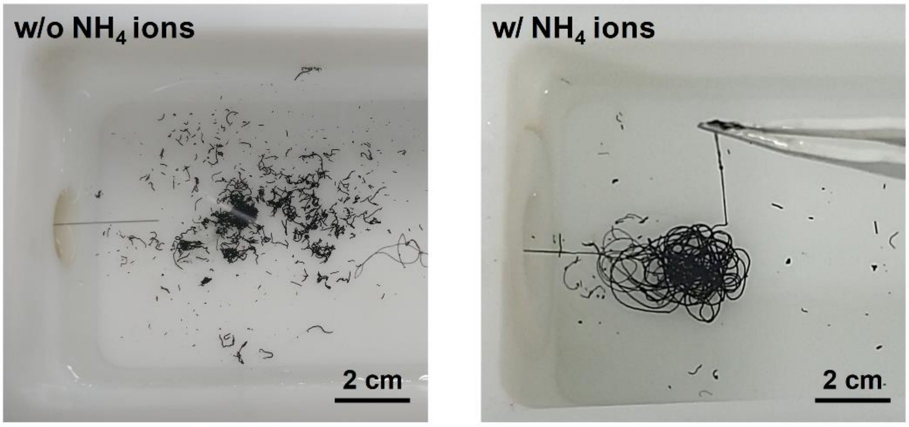

Supplementary Figure S5. The introduction of ammonium ions increased the molecular interactions between the MXene sheets.

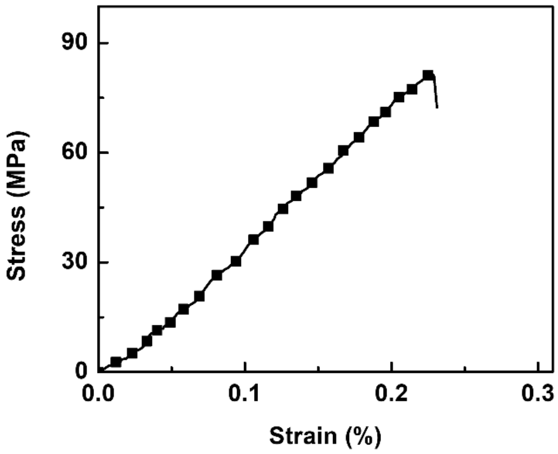

Supplementary Figure S6. Stress-Strain curve of the pure MXene fibers.

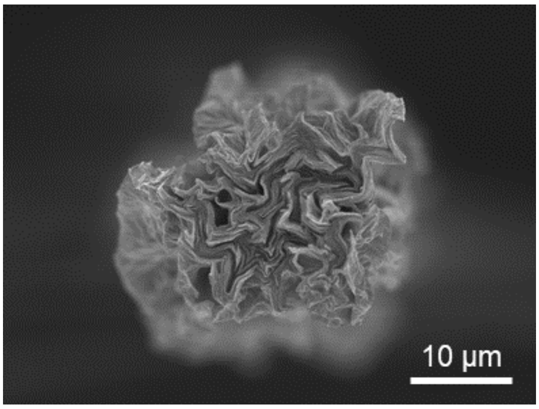

Supplementary Figure S7. A cross-section SEM image of a pure MXene fiber.

Supplementary References

1. Seyedin, S., Yanza, E. R. S. & Razal, J. M. Knittable energy storing fiber with high volumetric performance made from predominantly MXene nanosheets. *J. Mater. Chem. A* **5,** 24076-24082 (2017).
2. Yang, Q. et al. MXene/graphene hybrid fibers for high performance flexible supercapacitors. *J. Mater. Chem. A* **5,** 22113-22119 (2017).
3. Wang, Z. et al. High-performance biscrolled MXene/carbon nanotube yarn supercapacitors. *Small* **14,** 1802225 (2018).
4. Zhang, J. et al. Highly conductive $Ti_3C_2T_x$ MXene hybrid fibers for flexible and elastic fiber-shaped supercapacitors. *Small* **15,** 1804732 (2019).
