# Supplementary material for: Large-scale wet-spinning of highly electroconductive MXene fibers
Source: Nat Commun. 2020 Jun 4;11:2825. doi: 10.1038/s41467-020-16671-1 (PMC7272396; doi:10.1038/s41467-020-16671-1)
Supplement: Supplementary file 2 — Peer Review File [file 41467_2020_16671_MOESM2_ESM.docx]

Peer Review Information

**Journal:** Nature Communications

**Manuscript title:** Large-scale Wet-spinning of Highly Electroconductive MXene Fibers

**Corresponding author name(s):** Prof Tae Hee Han

**Reviewer comments & decisions:**

**Reviewer comments, first version:**

Reviewers' comments:

Reviewer #2 (Remarks to the Author):

Han et al. prepared pure Ti3C2Tx (All-MXene) fibers via wet-spinning by using H2O as a solvent and NH4Cl and NH4OH as a coagulator, and the resultant flexible, meter-long MXene fibers showed a very high electrical conductivity of 7,713 S cm-1, and they were successfully switched on a white LED light and were integrated into earphone wires to transmit electrical signals. This work is very innovative. However, several points still need to be addressed before the manuscript can be accepted:
1. How to prove that the aluminum in the interlayer is completely etched? How the MXene sheets are obtained? Corresponding XRD patterns are very necessary to provide.
2. As is known to all, MXene nanosheets are very easy to be oxidized, and it can see that the peak strength of TiO2 is relatively high in the O 1s XPS spectrum, so how to explain this phenomenon? In addition, please provide the Ti 2p XPS spectrum of the MXene fiber.
3. You said that the MXene in dispersion does not sink well through centrifugation when the pH of the solution is almost neutral, why? some detailed discussions are needed.
4. Conductive atomic force microscopy (C-AFM) showed that the MXene sheets were highly electrically conductive，so the MXene fiber can also be measured by C-AFM? How the high conductivity of the fiber is obtained?
5. We know that the size of MXene nanosheets is relatively small, by rights, it is difficult to directly prepare flexible fibers like graphene. Young's modulus is the physical quantity that represents the elasticity of the material itself. However, the fiber prepared is mainly used for flexible devices, so the toughness of the fiber is needed to be analyzed, the stress-strain curve of MXene fiber may be needed to further demonstrate its mechanical properties.
6. The language and structure of the article need to be further improved to meet the requirements of journals.


Reviewer #3 (Remarks to the Author):

The contribution of Han et al. is very interesting. The manuscript is well-written and presented. The material looks very promising and the synthetic approach seems feasible and easy to carry.
This work will surely be interesting for many researchers working in the field of materials and material manipulation.
I have only few questions:
1: how was the deconvolution of the O band (XPS, fig. 2) made? the band is pretty broad and I wonder whether such fine attribution can be confirmed with other experimental technique. In other words, was the surface composition and the composition of the material characterized by other method? a related question concerns the purity of the material. Are there any side product? Can the Al of the precursor be completely washed away?
2: the authors stress the large scale production, which is also visible from the video provided. Could be the yield quantified?
3.can the thickness of the fibers be adjusted? If so, has this an effect on final performances. This is just a curiosity of mine.

**Author rebuttal, first version:**

***1. Reviewer #1 expressed concerns to the editor about the importance of large flakes for spinnability, the particular coagulation bath, and MXene liquid crystals.***

**Our Response:** We do agree that the size of MXene flakes is a very critical parameter for spinnability. In our process, we could make _Ti3C2T_*_x_* MXene flakes as large as 100 μm in their lateral size. An average flake size was 2.26 ± 0.95 μm, as shown in **Fig. S1b,** and **c**. To give the direct impression for the size of MXene flakes, we updated the previous submission by modifying the flake area (μm^2^) to lateral size (μm) throughout the revised manuscript.

1

**Fig. S1b,** and **c**. **b** SEM image of fully exfoliated MXene sheets. **c** Lateral size distribution of MXene sheets. The distribution of the sheet sizes was obtained by measuring 635 sheets in SEM images. The mean lateral size of the sheets was 2.26 ± 0.95 µm.

(Modified in manuscript, page 3, line 25)

SEM images of fully exfoliated MXene monolayers showed an average lateral size of 2.26 ±

0.95 μm (**Fig. 1b** and **S1b, c**).

***2. Reviewer #1 also questions how cross-sectional area of the fibers and electrical conductivity are calculated and has concerns about lack of a stress-strain curve and assessment of recent literature.***

**Our Response:**

Cross-sectional area and electrical conductivity

The cross-sectional area was directly obtained by measuring the cross-section SEM image of MXene fibers (**Fig. S7**). The electrical conductivity was obtained by calculating the electrical resistance measured using a multimeter (DMM 7510 1/2, Keithley Instruments, USA) *via* the four-point probe method. The distance between four-electrodes was ≈ 0.4 mm. The conductivity (*ρ*) of MXene fiber was calculated by following equation^46^.

*ird^2^R*

| *ߩ =* |  |
| --- | --- |
|  | *4L* |

where d is the diameter, R is the electrical resistance, and L denotes the length of the fiber. The diameter of fiber was calculated, based on the cross-sectional area measured from SEM.

(Newly added reference)

46. Rebouillat et al. Measuring the Electrical Conductivity of Single Fibres, *Int. J. Electrochem.*

*Sci.* **6**, 5731–5740 (2011).

2

**Fig. S7**. A cross-section SEM image of a pure MXene fiber.

Stress-strain curve

The stress-strain curve of MXene fiber is presented in **Fig. S6**.

**Stress (MPa)**

**90**

**60**

**30**

**0**

**0.0	0.1	0.2	0.3**

**Strain (%)**

**Fig. S6**. Strain-Stress curve of the pure MXene fibers.

Assessment of recent literature

Recently, previous works on the fabrication of MXene fibers based on wet-spinning and electrospinning have used additives such as polymeric matrix binder and graphene oxide.^20,21^ However, the intrinsically high electrical conductivity of pure _Ti3C2T_*_x_* MXene (up to 9,880 S cm^-1^ for spray-casted films) is lowered compared with that of MXene composites with rGO (72, 290 S cm-1),21,22 CNT fiber (26 S cm^-1^),^23^ and PEDOT: PSS (1,489 S cm^-1^),^24^ showing that the conductivity of MXene is not fully utilized in the fiber form.

References in main text

20. Jiang, C. et al. All-electrospun flexible triboelectric nanogenerator based on metallic MXene nanosheets. *Nano Energy* **59**, 268–276 (2019).
21. Seyedin, S., Yanza, E. R. S. & Razal, J. M. Knittable energy storing fiber with high volumetric performance made from predominantly MXene nanosheets. *J. Mater. Chem. A* **5**, 24076–24082 (2017).
22. Yang, Q. et al. MXene/graphene hybrid fibers for high performance flexible

3

supercapacitors. *J. Mater. Chem. A* **5**, 22113–22119 (2017).

23. Wang, Z. et al. High-Performance Biscrolled MXene/Carbon Nanotube Yarn Supercapacitors. *Small* **14**, e1802225 (2018).
24. Zhang, J. et al. Highly Conductive Ti3C2Tx MXene Hybrid Fibers for Flexible and Elastic Fiber-Shaped Supercapacitors. *Small* **15**, e1804732 (2019).

(Modified in manuscript, page 8, line 21)

The electrical conductivity of the MXene fibers was measured using a multimeter (DMM 7510 1/2, Keithley Instruments, USA) via the four-point probe method. Four electrodes were separated by a distance of 0.4 mm. Since the conductivity of MXene fibers is highly affected by the atmospheric humidity, the measurement was made in a dry chamber. The conductivity (ρ) of a single fiber was calculated by the following equation:^46^

*ߨ݀ଶܴ*

| *ߩ =* |  |
| --- | --- |
|  | *4ܮ* |

where d is the diameter, R is the electrical resistance, and L is the length of the fiber. The diameter of the fiber was calculated based on the cross-sectional area measured in the scanning electron microscope (**Fig. S7**)

(Newly added reference)

46. Rebouillat et al. Measuring the Electrical Conductivity of Single Fibres, *Int. J. Electrochem.*

*Sci.* **6**, 5731-5740 (2011).

(Modified in manuscript, page 5, line 32)

**Fig. 4** compares the electrical conductivity and Young’s modulus of our fabricated MXene fibers with those of MXene hybrid fibers and graphene fibers fabricated in previous studies (**Fig. S6** and **Table S2**).

**3. *Reviewer #1 and Reviewer #3 have reservations about product purity.***

**Our Response:** Thank you so much for the comment. Firstly, product purity was examined by XPS study. In the wide scan XPS spectrum of MXene (**Fig. S3a**), the distinctive peaks for residual impurities are not observed. Atomic information is also summarized in **Table S1**. The amount of the detected Al was extremely less without any other impurities.

In addition, the XRD spectra of the MAX phase (Ti3AlC2) and MXene flakes (Ti3C2T*x*) (**Fig. S2c**) give an important information for the purity of MXene flakes. The typical MAX phase peaks were not observed after the etching of fully exfoliated MXene flakes, as shown in

4

**Fig. S2c**. The lowest angle (002) peak at 9.62° shifts to 7.14° after etching (Ti3AlC2 powder), and (104) peak at 39.06^o^ is removed, which confirms the complete etching of Al layers. Thus, we conclude that the synthesized MXene flakes are extremely pure. The manuscript was modified by addressing the purity of MXene flakes.

**Intensity (a. u.)**

**1200	900	600	300	0**

**MAX Phase MXene**

O 1s

Al 2p

F 1s

Ti 2p

C 1s

**Binding energy (eV)**

**Fig. S3a**. XPS survey of MXene _(Ti3C2T_*_x_*_)_ and MAX phase (Ti3AlC2).
**Table S1**. Atomic percent of MXene _(Ti3C2T_*_x_*_)_ and MAX phase (Ti3AlC2).

| Sample | Ti 2p | Al 2p | C 1s | O 1s | F 1s |
| --- | --- | --- | --- | --- | --- |
| Ti3AlC2 | 8.48 | 11.24 | 36.41 | 43.75 | 0.12 |
| Ti3C2T*x* | 29.94 | 0.11 | 39.09 | 17.92 | 12.94 |

**Fig. S2c**. XRD patterns of MXene _(Ti3C2T_*_x_*_)_ and MAX phase (Ti3AlC2).

(Modified in manuscript, page 4, line 11)

The deconvoluted C 1s, O 1s, and Ti 2p XPS peaks demonstrated that inherent termination

groups, such as C-Ti-F_x_, C-Ti-(OH)_x_, and C-Ti-O_x_, existed on the surface of the MXene and

5

were likely introduced during the Al etching of the MAX crystals (**Fig. 2a, b, Fig. S2a-c**).

(Newly added reference)

27. F. Liu et al. Preparation of _Ti3C2_ and Ti2C MXenes by fluoride salts etching and methane

adsorptive properties. *Appl. Surf. Sci.* **416**, 781–789 (2017).

**Comments from Reviewer #2**

*Han et al. prepared pure _Ti3C2Tx_ (All-MXene) fibers via wet-spinning by using H2O as a solvent and NH4Cl and NH4OH as a coagulator, and the resultant flexible, meter-long MXene fibers showed a very high electrical conductivity of 7,713 S cm^-1^, and they were successfully switched on a white LED light and were integrated into earphone wires to transmit electrical signals. This work is very innovative. However, several points still need to be addressed before the manuscript can be accepted:*

***1. How to prove that the aluminum in the interlayer is completely etched? How the MXene sheets are obtained? Corresponding XRD patterns are very necessary to provide.***

**Our response:** Thank you so much for the comment and important suggestions. The MXene sheets were synthesized by etching the Al layers in the MAX phase using LiF and HCl. As suggested, XRD analysis was performed to confirm the etching of the Al layers (**Fig. S2c**).^27^ The lowest angle (002) peak at 9.62° shifts to 7.14° after the etching Al layers in Ti3AlC2 . The (104) peak at 39.06^o^ is simultaneously removed, confirming the complete etching of Al layers. We have added the XRD in the supplementary information, as **Fig. S2c** and discussion in the revised manuscript.

In addition, the distinctive peaks for residual impurities are not observed in the wide scan XPS spectrum of MXene (**Fig. S2a**). Atomic information is also summarized in **Table S1**. The amount of the detected Al is extremely less. This shows that MXene flakes were successfully delaminated after the complete removal of Al layers in the MAX phase.

6

**Fig. S2c**. XRD patterns of MXene _(Ti3C2T_*_x_*_)_ and MAX phase (Ti3AlC2).

**Intensity (a. u.)**

**1200	900	600	300	0**

**MAX Phase MXene**

O 1s

Al 2p

F 1s

Ti 2p

C 1s

**Binding energy (eV)**

**Fig. S3a**. XPS spectra of MXene _(Ti3C2T_*_x_*_)_ and MAX phase (Ti3AlC2).

**Table S1**. Atomic percents of components in the MXene _(Ti3C2T_*_x_*_)_ and MAX phase (Ti3AlC2).

| Sample | Ti 2p | Al 2p | C 1s | O 1s | F 1s |
| --- | --- | --- | --- | --- | --- |
| Ti3AlC2 | 8.48 | 11.24 | 36.41 | 43.75 | 0.12 |
| Ti3C2T*x* | 29.94 | 0.11 | 39.09 | 17.92 | 12.94 |

(Modified in manuscript, page 4, line 7)

The XRD pattern and the atomic percent of MXene confirmed the complete etching of the Al

layer (**Fig. S2c** and **Table S1**).^27^

(Newly added reference)

27. F. Liu et al. Preparation of _Ti3C2_ and Ti2C MXenes by fluoride salts etching and methane

adsorptive properties. *Appl. Surf. Sci.* **416**, 781-789 (2017).

7

2. ***As is known to all, MXene nanosheets are very easy to be oxidized, and it can see that the peak strength of TiO2 is relatively high in the O 1s XPS spectrum, so how to explain this phenomenon? In addition, please provide the Ti 2p XPS spectrum of the MXene fiber.***

**Our response:** Thank you for seeking our attention to the important discussion on the XPS of MXene. The MXene sheets were synthesized by etching the Al layers from the MAX phase using strong acid containing HCl and LiF. During the etching process of the Al layer, the formation of TiO2 on MXene is inevitable^R1^. In this work, with controlled synthesis conditions, the peak intensity of TiO2 is relatively low in the Ti 2p XPS spectrum (**Fig. S3b**). Moreover, from the XPS results, it is observed that the terminal groups such as C-Ti-F_x_, C-Ti-O_x_, and C-Ti-(OH)2 are more prominent than TiO2. We have added the Ti 2p XPS in the supplementary information, as **Fig. S3b** and discussion in the revised manuscript.

**Binding energy (eV)**

**465	460	455**

**Intensity (a. u.)**

**Ti 2p**

Ti^3+^

TiO_2_

Ti^2+^

Ti-C

**Fig. S3b**. Ti 2p XPS spectrum of the MXene.

Reference in response letter

R1. J. Halim et al. X-ray photoelectron spectroscopy of select multi-layered transition metal carbides (MXenes), *Appl. Surf. Sci*. **362**, 406–417 (2016).

(Modified in manuscript, page 4, line 12)

The deconvoluted C 1s, O 1s, and Ti 2p XPS peaks demonstrated that inherent termination groups, such as C-Ti-F_x_, C-Ti-(OH)_x_, and C-Ti-O_x_, existed on the surface of the MXene and were likely introduced during the Al etching of the MAX crystals (**Fig. 2a, b, Fig. S2a-c**).

2. ***You said that the MXene in dispersion does not sink well through centrifugation when the*** 8

***pH of the solution is almost neutral, why? some detailed discussions are needed.***

**Our response:** Thank you so much for this important comment. After the etching process of the Al layers in the MAX phase, the obtained MXene dispersion contained unexfoliated MXene and residual acid.^25^ The acidic product was washed copiously with deionized water using a centrifuge until pH ≥ 6. We found that unexfoliated MXene flakes and acidic supernatant were efficiently removed during this step. The surface of MXene in acid conditions has poor zeta potential, which causes it to settle easily (**Fig. 2c**). However, after the elimination of reaction products, the negative zeta potential increased on the surface of MXene to obtain a well-dispersed MXene solution. These phenomena about MXene dispersion are in well accordance with the previous reports. ^R2^ As suggested, the manuscript was modified as below.

References in main text and response letter

25. Alhabeb, M. et al. Guidelines for Synthesis and Processing of Two-Dimensional Titanium

Carbide _(Ti3C2Tx_ MXene), *Chem. Mater.* **29**, 7633–7644 (2017).

R2. Li et al. On the Synthesis of Low-Cost, Titanium-Based MXenes, *J. Ceram. Sci. Technol.* **7**,

301–306 (2016).

(Modified in manuscript, page 12, line 18)

To separate the MXene dispersion from the acid, it was washed with water using a centrifuge until the pH reached 6. As the pH of the solution was almost neutral, the MXene in the dispersion did not sink well through centrifugation due to an increase in the negative zeta potential of their surface.

***4. Conductive atomic force microscopy (C-AFM) showed that the MXene sheets were highly electrically conductive, so the MXene fiber can also be measured by C-AFM? How the high conductivity of the fiber is obtained?***

**Our response:** Thank you so much for the comment. Due to the highly rugged surface nature of MXene fibers, the C-AFM was not suitable to measure the electrical conductivity of MXene fiber. Herein, this work electrical conductivity of MXene fiber was measured using a multimeter (DMM 7510 1/2, Keithley Instruments, USA) via the four-point probe method^46^. The distance between four-electrodes was ≈ 0.4 mm. Since the conductivity of MXene fiber is profoundly affected by atmospheric humidity, the measurement was performed in a dry chamber. The conductivity (ρ) of single fiber was calculated by following equation^46^.

9

| *ߩ =* | *ߨ݀^2^ܴ* |
| --- | --- |
|  | *4ܮ* |

where d is the diameter, R is the electrical resistance, and L denotes the length of the fiber. The electrical conductivities under atmospheric humidity and the dry condition are shown in **Fig. R1**. The MXene fibers under atmospheric humidity (21 % RH) exhibits electrical conductivity of 6883 S cm^-1^. However, MXene fibers under dry condition (4 % RH) display an ultrahigh electrical conductivity of 7,713 S cm^-1^.

(Modified in manuscript, page 13, line 23)

The electrical conductivity of the MXene fibers was measured using a multimeter (DMM 7510 1/2, Keithley Instruments, USA) via the four-point probe method. Four electrodes were separated by a distance of 0.4 mm. Since the conductivity of MXene fibers is highly affected by the atmospheric humidity, the measurement was made in a dry chamber. The conductivity (ρ) of a single fiber was calculated by the following equation:^46^

*ߨ݀^2^ܴ*

| *ߩ =* |  |
| --- | --- |
|  | *4ܮ* |

where d is the diameter, R is the electrical resistance, and L is the length of the fiber. The diameter of the fiber was calculated based on the cross-sectional area measured in the scanning electron microscope (**Fig. S7**)

(Newly added reference)

46. Rebouillat et al. Measuring the Electrical Conductivity of Single Fibres, *Int. J. Electrochem.*

*Sci.* **6**, 5731–5740 (2011).

| **8.0x10^3^**  **7.0x10^3^**  **6.0x10^3^** |  |
| --- | --- |

**21**

**Electrical conductivity (S cm-1)**

**(Atmospheric humidity)**

**4**

**(In dry**

**chamber)**

**Humidity (% RH)**

**Fig. R1**. Measured electrical conductivity under different humidity conditions.

10

***5. We know that the size of MXene nanosheets is relatively small, by rights, it is difficult to directly prepare flexible fibers like graphene. Young's modulus is the physical quantity that represents the elasticity of the material itself. However, the fiber prepared is mainly used for flexible devices, so the toughness of the fiber is needed to be analyzed, the stress-strain curve of MXene fiber may be needed to further demonstrate its mechanical properties.***

**Our response:** Thank you so much for your valuable suggestions. As commented, it is extremely difficult to wet-spun flexible fibers having a small size without using matrix materials such as polymeric binders and graphene oxide so far. To overcome this, herein this work, we have successfully prepared large size MXene sheets for wet-spinning flexible fibers without binders. To demonstrate the mechanical performance of the prepared fibers, the additional tensile test of MXene fiber was performed. We tested several samples with a length of 10 mm, and the calculated toughness was 0.091 ± 0.011 μm^2^. The mechanical properties were updated as below.

**Stress (MPa)**

**90**

**60**

**30**

**0**

**0.0	0.1	0.2	0.3**

**Strain (%)**

**Fig. S6**. Strain-Stress curves of the pure MXene fibers.

(Modified in supplementary information, page 2)

**Table S2**. Comparison of the performances of different _Ti3C2T_*_x_* MXene fibers.

|  | **Electrical** |  |  | **Young’s** |  |
| --- | --- | --- | --- | --- | --- |
| **Sample** | **conductivity** | **Tensile Strength** | **Tensile Elongation** | **Modulus** | **Toughness** |
|  | **(S cm^-1^)** | **(MPa)** | **(%)** | **(GPa)** | **(MJ m^-3^)** |
| Pure MXene |  |  |  |  |  |
|  | 7713 ± 110 | 63.9 ± 13.1 | 0.22 ± 0.05 | 29.6 ± 5.1 | 0.091 ± 0.011 |
| Fiber |  |  |  |  |  |

***6. The language and structure of the article need to be further improved to meet the requirements of journals.***

11

**Our response:** Thank you so much for your valuable suggestions. The structure of the manuscript was modified according to the guidelines of *Nature Communications*. The language of the manuscript was modified and improved by requesting for the proofreading from the *Nature Research Editing Service*.

**Comments from Reviewer 3**

*The contribution of Han et al. is very interesting. The manuscript is well-written and presented. The material looks very promising and the synthetic approach seems feasible and easy to carry. This work will surely be interesting for many researchers working in the field of materials and material manipulation. I have only few questions:*

*1. How was the deconvolution of the O band (XPS, fig. 2) made? the band is pretty broad, and I wonder whether such fine attribution can be confirmed with other experimental technique. In other words, was the surface composition and the composition of the material characterized by other method? a related question concerns the purity of the material. Are there any side product? Can the Al of the precursor be completely washed away?*

**Our response:** We appreciate your valuable comments. The O 1s XPS peaks were deconvoluted using Gaussian-Lorentzian distribution curves. We do agree that the O 1s band looks broad.

12

However, compared to previous reports R1, R3, R4, our result did not appear to be broad (**Fig. R2**).

The deconvoluted peaks were found to be in accordance with previous XPS studies of Ti3C2T*x* MXenes.R1, R3, R4

**Fig. R2**. XPS O 1s spectra of _Ti3C2T_*_x_* from **a** our work **b** ref. R3.

The XPS survey shows the presence of Ti, O, C, and F elements (**Fig. R3a**), while the Al peak was completely removed after etching. Also, the deconvoluted Al 2p XPS spectrum (**Fig. R3b, c**) shows the absence of peaks confirming the complete elimination of Al layers^25^. The XPS results show the presence of terminal groups such as C-Ti-F_x_, C-Ti-O_x_, and C-Ti-(OH)2 with minor TiO2 peak (**Fig. 2a, b**).

**Fig. S3**. **a** XPS survey and XPS high resolution spectra of **b** Ti 2p, and **c** Al 2p of the MXene.

The XRD patterns (**Fig. S2c**) were also provided to examine the structure of MXene flakes. The XRD analysis was performed after etching (Ti3AlC2 powder). The lowest angle (002) peak at 9.62° shifts to 7.14° after etching (Ti3AlC2 powder), and (104) peak at 39.06^o^ is removed, which confirms the complete etching of Al layers.

13

**Fig. S2c**. XRD patterns of MXene _(Ti3C2T_*_x_*_)_ and MAX phase (Ti3AlC2).

We have added the XPS survey (**Fig. S3a**), XPS high resolution spectrum of Ti 2p (**Fig. S3b**), Al 2p (**Fig. S3c**), and XRD (**Fig. S2c**) in the supplementary information and discussion in the revised manuscript.

References in main text and response letter

25. Alhabeb, M. et al. Guidelines for Synthesis and Processing of Two-Dimensional Titanium

Carbide _(Ti3C2T_*_x_* MXene), *Chem. Mater.* **29**, 7633−7644 (2017).

R3. Shah et al. Template-free 3D titanium carbide (Ti3C2Tx) MXene particles crumpled by capillary forces, *Chem. Commun*., **53**, 400−403 (2017).
R3. Han, M. et al. Ti3C2 MXenes with Modified Surface for High-Performance Electromagnetic Absorption and Shielding in the X-Band, *ACS Appl. Mater. Interfaces* **8**, 21011−21019 (2016).

(Modified in manuscript, page 4, line 6)

The XRD pattern and the atomic percent of MXene confirmed the complete etching of the Al

layer (**Fig. S2c** and **Table S1**).

(Modified in manuscript, page 4, line 11)

The chemical functionalities of the exfoliated MXene sheets were further examined via X-ray photoelectron spectroscopy (XPS). The deconvoluted C 1s, O 1s, and Ti 2p XPS peaks demonstrated that inherent termination groups, such as C-Ti-F_x_, C-Ti-(OH)_x_, and C-Ti-O_x_, existed on the surface of the MXene and were likely introduced during the Al etching of the MAX crystals (**Fig. 2a, b, Fig. S2a-c**).

14

2. *The authors stress the large scale production, which is also visible from the video provided. Could be the yield quantified?*

**Our response:** Thank you very much for your interesting question. The production yield is very important for the practical utilization of our fibers. The fiber in the video were produced at a rate of 2.8 cm sec^-1^. And we extrude about 1,400 cm per 1 mL of MXene (25 mg mL^-1^). Currently, we have successfully produced 1.2 meter-long continuous MXene fibers using a simple wet-spinning method without breakage. We are continuously developing our process, and the current value has been much more improved up to 3.2 m since our submission.

3. *Can the thickness of the fibers be adjusted? If so, has this an effect on final performances. This is just a curiosity of mine.*

**Our response:** We appreciate your thoughtful query. As commented, the thickness of fiber has a huge effect on the final performance. In the case, the thinner fiber, the values of electrical conductivity, and mechanical properties can be increased due to the low cross-sectional area values. However, poor physical performance, such as low carrying current, load amount, along with stress deteriorates, which is not suitable for practical applications. Therefore, we have fabricated thicker fiber for multipurpose applications. As mentioned in the manuscript, during the wet-spinning process, the fibers are aligned by the shear stress induced by the pulling rate. We found that the core part of the thick fiber is not fully aligned, resulting in weak mechanical and low electrical properties.^11^ Thus, optimizing the thickness of the fibers is crucial for practical applications.

Reference in main text

11. Park, H. et al. Dynamic assembly of liquid crystalline graphene oxide gel fibers for ion

transport. *Sci. Adv.* **4**, eaau2104 (2018).

**Reviewer comments, second version:**

Reviewer #2 (Remarks to the Author):

Authors have corrected the comments from reviewers point by point, I recommend this manuscript to be published.
Aminor comment:
About the purity of MAX and Ti3C2Tx phases, authors have supported their XRD patterns and XPS spectra.However, in their XPS spectra, O1s 43.75% for MAX phase is very larger than 17.92% of Ti3C2Tx, authors should give explain why hier in MAX phase.


Reviewer #3 (Remarks to the Author):

The authors carefully replied comments from the reviewers.
The answers and explanations seem satisfying.
There is one more comment to be taken into account before acceptance: the authors should clearly state, when discussing XPS data, that those refer only to the surface and cannot be extended to the whole material (this is for instance, when they discuss purity).
in a similar way, when discussing absence/presence of phases from XRD data, it should be specified that this applies only to crystalline phases. residual amorphous phases can be hardly detected by x-rays diffraction.

**Author rebuttal, second version:**

*Authors have corrected the comments from reviewers point by point, I recommend this manuscript to be published.*

***1. About the purity of MAX and Ti_3_C_2_T_x_ phases, authors have supported their XRD patterns and XPS spectra. However, in their XPS spectra, O1s 43.75% for MAX phase is very larger than 17.92% of Ti3C2Tx, authors should give explain why hier in MAX phase.***

**Our Response:**

As commented, our MAX phase has higher oxygen content (43.75%) than that of MXene (17.92%). It might be due to the formation of aluminum oxide (Al_2_O_3_) on the MAX phase.^R1,2^ Thus, the atomic content of oxygen becomes high, more than 30% (Figure R1).^R3,4^ To confirm the oxidation effect of the MAX phase, Argon-ion beam etching was carried out on the surface of MAX phase (Figure R2). As summarized in Figure R1 and R2, the amount of Al_2_O_3_ was considerably reduced after ion-beam etching. On the other hand, the Al content of the MAX phase was removed after selective etching using HCl and LiF (Fig. S3c and Figure R3). Therefore, it was confirmed that the higher content of O1s in the MAX phase was due to the formation of Al_2_O_3_ on the edge and surface of the MAX phase.

Reference

R1. Tallman, D. J., Anasori, B., & Barsoum, M. W. A critical review of the oxidation of Ti_2_AlC, Ti_3_AlC_2_ and Cr_2_AlC in air. *Mater. Res.* **1**, 115–125 (2013).

R2. Barsoum, M. W. MAX phases: properties of machinable ternary carbides and nitrides. John Wiley & Sons (2013).

R3. Zhu, M. et al. Comparison of corrosion behavior of Ti_3_SiC_2_ and Ti_3_AlC_2_ in NaCl solutions with Ti, *Ceram. Int.* **43**, 5708–5714 (2017).

R4. Zhang, Z. et al. Probing the oxidation behavior of Ti_2_AlC MAX phase powders between 200 and 1000 °C, *J. Eur. Ceram. Soc.* **37**, 43–51 (2017).

**Figure R1.** O 1s and Al 2p XPS spectra of the MAX phase.

**Figure R2.** O 1s XPS spectra of surface and surface etched MAX phase.

**Figure R3.** Al 2p XPS spectra of MAX phase and MXene.

**Comments from Reviewer #3**

*The authors carefully replied comments from the reviewers. The answers and explanations seem satisfying.*

***1. There is one more comment to be taken into account before acceptance: the authors should clearly state, when discussing XPS data, that those refer only to the surface and cannot be extended to the whole material (this is for instance, when they discuss purity). in a similar way, when discussing absence/presence of phases from XRD data, it should be specified that this applies only to crystalline phases. residual amorphous phases can be hardly detected by x-rays diffraction.***

**Our response:**

As pointed out, the surface analysis tools for the multiple atomic layers give limited information about the whole material. As discussed in the main text, the disappearance of Al layers was characterized by XRD because the information for interlayer distance in the MAX phase is changed at the assembled structures of a single layer of MXene^R5-7^.

Besides XRD characterization, further, we performed the XPS experiment to get the direct information about the existence of Al along the z-direction of Ti_3_C_2_T*_x_* MXene films by applying the sequential etching of MXene film with the focused Ar ion-beam^R8^.

First, the surface of the MXene film was surveyed with XPS. Then the film was focused with the Ar-ion beam. After etching, the XPS analysis was again performed on the films. These steps were repeated 10 times. The etching condition was 2kV accelerated high-energy Argon ion beam with 1.5 mm of X and Y raster^R9^. The etched depth was about 5 μm, which is confirmed by using an Alpha-step measurement (Figure R4).

As demonstrated in Figure R5, the XPS depth profile provides information about the elemental distribution at a different depth. Titanium (Ti), carbon (C), oxygen (O), and fluorine (F) in the Ti_3_C_2_T*_x_* MXene film was observed in the wide scan of XPS (Figure R5a). The XPS spectra of Al 2p (Figure R5b) indicates that the Al layer in the MAX phase disappears, manifesting the successful removal of Al.

References

R5. Lee, E. et al. Room temperature gas sensing of two-dimensional titanium carbide (MXene). *ACS Appl. Mater. Inter.* **9**, 37184–37190 (2017).

R6. Mashtalir, O. et al. Intercalation and delamination of layered carbides and carbonitrides. *Nat. commun.* **4**, 1–7 (2013).

R7. Hu, M. et al. Surface functional groups and interlayer water determine the electrochemical capacitance of Ti3C2 T x MXene. *ACS Nano* **12**, 3578–3586 (2018).

R8. Gilbert, J. B. et al. Determination of lithium-ion distributions in nanostructured block polymer electrolyte thin films by X-ray photoelectron spectroscopy depth profiling. *ACS Nano* **9**, 512–520 (2015).

R9. Datta, M. et al. Characterization of transpassive films on nickel by sputter profiling and angle resolved AES/XPS. *Appl. Surf.* **18**, 299–314 (1984).

**Figure R4.** The surface profile of surface etched MXene film obtained from the alpha step profiler.

**Figure R5.** XPS depth profile of Ti_3_C_2_T*_x_* MXene film. XPS spectra of (a) survey, and (b) Al 2p of Ti_3_C_2_T*_x_* MXene film during Ar-ion beam etching.

**Reviewer comments, third version:**

**Author rebuttal, third version:**
